# Microglial dynamics and ferroptosis induction in human iPSC‐derived neuron–astrocyte–microglia tri‐cultures

**DOI:** 10.1002/2211-5463.70182

**Published:** 2026-01-14

**Authors:** Hongmei Lisa Li, Hiroko Ohmiya, Sou Sakamoto, Masato Yugami, Akiko Oki, Makoto Furusawa, Yan Ling

**Affiliations:** ^1^ Neuroscience Translational Medicine, Neuroscience Drug Discovery Unit, Research Takeda Pharmaceutical Company Fujisawa Japan; ^2^ Present address: Takeda APAC Pharmaceuticals R&D Ltd Shanghai China

**Keywords:** ferroptosis, iPSC tri‐culture, single‐cell RNA‐seq, transcriptomics

## Abstract

The dynamics of microglial activity within neuron–astrocyte–microglia tri‐cultures derived from human induced pluripotent stem cells (iPSCs) present a complex interplay and offer an opportunity to obtain new insights into neuron**–**glia interactions. Iron‐laden microglia, correlating with functional changes, represent a key pathological feature of Alzheimer's disease (AD). This study characterized the cellular crosstalk and transcriptional states of microglia in tri‐cultures. Complement C3 can be detected in culture media when microglia are cocultured with neurons, and the addition of astrocytes in the coculture led to an increased amount of C3, indicating that the impact of glial interactions can be evaluated in this model system. We compared microglial gene expression profiles comprehensively in monoculture, coculture, and tri‐culture settings. Single‐cell RNA sequencing (scRNA‐seq) revealed various microglial states with gene expression changes associated with endocytosis and neuron‐related functions in tri‐culture settings, suggesting that microglial behavior is profoundly impacted by the presence of neurons and astrocytes. We assessed microglial responses to iron overload combined with the ferroptosis inducer RSL3 (a GPX4 inhibitor) in tri‐cultures. Microglial cell death was accompanied by ferritin heavy‐chain expression, indicating microglia ferroptosis. scRNA‐seq analyses highlighted alterations in pathways related to ferroptosis, stress response, and autophagy, indicating substantial shifts in microglial profiles upon iron perturbation. These findings underscore the necessity of using tri‐cultures as a model to capture certain degrees of complex cellular interactions occurring *in vivo*. These results offer critical insights for establishing *in vitro* models for therapeutic development of neurodegenerative diseases, including AD.

## Graphical abstract

A tri‐culture of iPSC‐derived neurons, astrocytes, and microglia treated with ferroptosis inducers as an Induced ferroptosis model was characterized by scRNA‐seq, cell survival, and cytokine release assays. This analysis revealed diverse microglial transcriptomic changes, indicating that the system captures key aspects of the complex cellular interactions between neurons and glial cells.

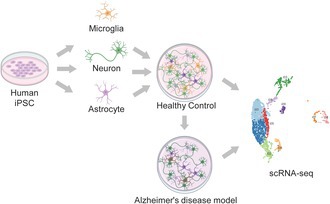

**Abbreviations**
AD: Alzheimer's disease
CNS: central nervous system
iPSC: induced pluripotent stem cell
FTH1: ferritin heavy chain 1
C3: complement component 3
NPC: neuronal progenitor cell
qPCR: quantitative polymerase chain reaction
scRNA‐seq: single‐cell RNA sequencing
GO: gene ontology

Microglia, the resident immune cells of the central nervous system (CNS), play a pivotal role in maintaining a homeostatic environment within the CNS [1, 2, 3]. During development and in the adult brain, microglial processes survey the local environment and exert CNS‐supportive functions via the following mechanisms: (1) clearance of metabolic waste and dead or dying neurons; (2) secretion of neurotrophic factors; (3) regulation of neuronal circuit plasticity and function via synaptic pruning and maintenance of synaptic contacts; and (4) limiting neurotoxic impact on neurons by isolating harmful substances [2]. These activities may be reengaged adaptively or maladaptively under aging and stress conditions, such as in neurodegeneration [4, 5, 6, 7]. In Alzheimer's disease (AD), microglial dysfunction is strongly implicated as a causal contributor to disease onset, as demonstrated by genome‐wide association study (GWAS) and epigenetic studies on AD [2]. As microglia reside in a complex microenvironment within the CNS parenchyma and communicate with other CNS and immune cells, the functional consequences of genetic variations occur in the context of multiple cellular interactions that ultimately lead to the accumulation of pathology. Conversely, the microglial state can also be influenced by neighboring cells and the presence of different pathologies. Thus, the establishment of an *in vitro* multicellular coculture system will allow us to evaluate the interplay between glia and neurons, thereby providing insights into how microglia impact neuronal health.

Advancements in human induced pluripotent stem cell (iPSC) technology provide a transformative approach to studying these interactions. iPSCs can differentiate into neurons, astrocytes, and microglia, offering a comprehensive model of the cellular environment in the brain [8]. Studer *et al*. reported a dynamic interplay between microglia and astrocytes indicated by changes in the secretion of C3 and TNF‐α [9]. Ryan *et al*. [10] reported microglia responses upon exposure to the ferroptosis inducer RSL3 in iPSC‐neuron, astrocyte, and microglia tri‐cultures. Our study focused on the detailed characterization of microglia within this tri‐culture system. Age‐related iron accumulation in the CNS and the corresponding increase in microglial ferritin expression suggest that microglia may be involved in iron sequestration in the aging brains [11, 12, 13]. Furthermore, it has been shown that degenerating microglia associated with aging‐related white matter injury express ferroptosis‐related genes [14]. We confirmed dynamic C3 secretion and recaptured microglial ferroptosis in response to ferroptotic stimuli. Furthermore, we utilized single‐cell RNA sequencing (scRNA‐seq) to compare microglial signatures across monoculture, neuron coculture, and tri‐culture, as well as to unravel the complex transcriptional landscapes and functional states of microglia in response to iron‐induced oxidative stress in tri‐culture conditions. These results provide evidence for a validated iPSC‐based tri‐culture system that can be modified to investigate microglial function and glia–neuron interactions in neurodegenerative diseases.

## Methods

### Preparation of human iPSC culture and human iPSC‐derived mono‐, co‐, and tri‐culture

Human iPSCs (clone 253G1, RRID:CVCL_B518) obtained from iPS Portal, Inc. (Kyoto, Japan) were cultured in StemFit AK02N medium (Ajinomoto, Chuo‐ku, Tokyo, Japan) coated with iMatrix‐511 (Nippi, Adachi, Tokyo, Japan). The iPSCs were fed daily and passaged every 3–4 days. The iPSCs (clone 253G1) were differentiated into cortical neuronal progenitor cells (NPCs) using a protocol modified from Shi *et al*. [15, 16]. NPC stocks were made on day 23. The same iPSCs (clone 253G1) were differentiated into microglia using the protocol originally described by Okuzono *et al*. [17]. Microglia stocks were obtained on day 22. iPSC monoculture: 253G1 iPSC‐derived microglia were thawed on day 0 and cultured until day 14. iPSC coculture of neurons and microglia: 253G1 iPSC‐derived microglia and 253G1 iPSC‐derived NPC cell stocks were thawed on day 0 and cultured until day 14. iPSC coculture of neurons and astrocytes: 253G1 iPSC‐derived microglia and iCell Astrocytes, thawed at day 0 with a seeding ratio of 3 : 1 and cultured until day 14. There were two types of iPSC tri‐cultures with neurons, astrocytes, and microglia: 253G1 iPSC‐derived NPC, 253G1 iPSC‐derived microglia, and iCell Astrocytes (C1037; FujiFilm Cellular Dynamics, Inc., FCDI), thawed at day 0 with a seeding ratio of 3 : 1 : 1 and cultured until day 14. Using these tri‐cultures, we performed C3 or IL‐8 secretion and scRNA‐seq analyses. The other was commercially available iCell GlutaNeurons (C1033), iCell Microglia (C1110), and iCell Astrocytes (all three cell types were from FCDI), thawed on day 0 at a seeding ratio of 4 : 1 : 1, and cultured until day 14. All cultures were routinely tested and found negative for mycoplasma contamination. Using these tri‐cultures, we performed Microglia and FTH1 kinetics. These tri‐cultures were poly l‐ornithine/fibronectin/laminin‐coated and maintained in FCDI iPSC tri‐culture medium. Half of the medium was exchanged every 3 or 4 days. The cells used in different assays come from the same differentiation protocols, and the replicates are independent seeding of cells.

### Immunocytochemistry (ICC)

The cells were fixed in 4% paraformaldehyde for 10 min at room temperature, permeabilized with 0.4% Triton X‐100 for 10 min, washed with 0.4% Triton X‐100 in phosphate‐buffered saline (PBS) for 5 min, and blocked with 1% goat serum in 0.4% Triton X‐100 in PBS for 30 min. The cells were incubated with primary antibodies overnight at 4 °C. Secondary antibodies (Thermo Fisher Scientific, Minato‐ku, Tokyo, Japan; Alexa Fluor 488 (A‐21202), 555 (A‐10042), and 647 (A‐21449)) were used at a 1 : 1000 dilution and incubated at room temperature for 120 min. Hoechst 33258 was used for nuclear staining. An Opera Phenix high‐content imaging system was used to quantify the number of microglial cells. The primary antibodies used were rabbit anti‐IBA1 polyclonal antibody (1 : 500, 019‐19 741; Wako, Naka‐ku, Hiroshima, Japan), mouse anti‐MAP2 monoclonal antibody (1 : 1000, MAB3418; Merck Millipore, Minato‐ku, Tokyo, Japan), chicken anti‐MAP2 polyclonal antibody (ab5392; Merck Millipore), chicken anti‐GFAP polyclonal antibody (1 : 500, Ab4674; Abcam, Chuo‐ku. Tokyo, Japan), and mouse anti‐human ferritin monoclonal antibody (1 : 500, MAB93541; R&D Systems, Chuo‐ku, Tokyo, Japan).

### Microglia and FTH1 kinetics

Data acquired using ICC were analyzed using Opera Phenix Harmony software to count marker expressions: MAP2 as a neuronal marker, GFAP as an astrocyte marker, IBA‐1 as a microglial marker, and FTH1 as an iron‐responsive marker. The unstimulated conditions at 0 h were each normalized to 100%.

### TNF‐α treatment

The iPSC coculture of neuron and astrocytes was treated for 72 h, depending on the experiment. The final concentrations were 100 ng·mL^−1^ TNF‐α. All the inhibitors were added as co‐treatments. The culture supernatants were collected for enzyme‐linked immunosorbent assay (ELISA) and Olink measurements and stored at −80 °C until use.

### Iron and RSL3 treatment

On day 14, the iPSC tri‐cultures were treated for 4–72 h, depending on the experiment. The final concentrations were 1 : 1000 DMSO (Sigma‐Aldrich, Meguro‐ku, Tokyo, Japan), 1600 μm FeSO4 (Sigma‐Aldrich), 1 μm RSL3 (Sigma‐Aldrich), 10 μm 4‐methyl‐2‐(4‐methylpiperazinyl) pyrimido [4,5‐*b*] benzothiazine (4‐MMPB) (Cayman Chemical, Bunkyo‐Ku, Tokyo, Japan), and 10 μm liproxstatin‐1(lip‐1) (Sigma‐Aldrich). All the inhibitors were added as co‐treatments. The culture supernatants were collected for enzyme‐linked immunosorbent assay (ELISA) and Olink measurements and stored at −80 °C until use.

### ELISA and Olink

C3 and IL‐8 levels in culture supernatants were analyzed using human C3 (Abcam) and IL‐8/CXCL8 ELISA kits (R&D Systems), respectively. Other cytokines in the culture supernatants were quantified using the Olink Target 48 Cytokine panel (Takara Bio, Kusatsu, Shiga, Japan). Statistical significance was calculated using Welch's *t*‐test.

### scRNA‐seq

iPSC tri‐culture cells were dissociated according to the protocol described in a previous report, with and without 4‐h treatment with iron and RSL3 [9]. Four hours after treatment, cells were washed with PBS twice. Cells were then treated with 0.25% trypsin + EDTA (T4049; Sigma‐Aldrich) + transcription/translation inhibitors (5 mg·mL^−1^ of actinomycin‐D (A1410; Sigma‐Aldrich) + 10 mm triplotide (T3652, Sigma‐Aldrich) + 27.1 mg·mL^−1^ of anisomycin (A9789; Sigma‐Aldrich)) for 6 min at 37 °C in a 5% CO_2_ incubator. Then, 1 volume of PBS + 2% FBS (A38400‐01; Gibco Inc., Billings, MT, USA) + transcription/translation inhibitors + 1 : 100 DNAse was added to quench. Cells were combined from two wells and put through 40‐mm cell strainers (352235; BD Falcon, Kasukabe City, Saitama Prefecture, Japan). Strained cell suspension was placed in a 1.7‐mL Eppendorf tube on ice. Cells were counted by ADAM‐MC. Cell suspensions were spun at 4 °C for 5 min at 528 ***g***. The supernatant was removed, and the cells were prepared using the Chromium Next GEM Single Cell Fixed RNA Sample Preparation Kit, 16 reactions (10× Genomics). The libraries were sequenced by NovaSeq (Illumina, Minato‐ku, Tokyo, Japan). Raw reads were aligned to the human reference genome GRCh38 using the CellRanger software version 7.1.0 (10× Genomics) [18]. After quantifying the expression of each mRNA using the CellRanger count pipeline, we used the SCANPY [19] library to remove outliers based on the pipeline proposed by Mathy *et al*. [20, 21]. The total number of aligned cells was 45 440, and after filtering, that number was 29 233. To manually predict cell types, we obtained marker genes from a previous study using CellMarker 2.0 [20, 22]. We calculated the average expression of a set of marker genes for each cell type and annotated each cell type based on the calculated values, following the integrative analysis workflow of Seurat [23]. After correcting batch effects using the Harmony algorithm [24], we identified clusters with a resolution of 0.5, using the Leiden algorithm [25, 26]. We applied Harmony to integrate scRNA‐seq datasets across modalities, such as technological platforms. The Harmony method is commonly used to integrate multiple scRNA‐seq datasets assayed with discrete technologies. To compare gene expression levels between groups, we used the R package edgeR [27]. To this end, we obtained pseudobulk datasets by summing the counts across cells within samples and clusters. To characterize significantly differentially expressed genes (false discovery rate (FDR) < 0.05), we used DAVID for the functional annotation [25]. We selected significantly enriched gene ontology terms in terms of biological processes after adjusting *P*‐values by calculating the FDR (FDR < 0.05).

## Results

### Characterization of human iPSC tri‐culture and distinct cellular and molecular changes in microglia comparing tri‐culture, coculture, and monoculture

**Fig. 1:**
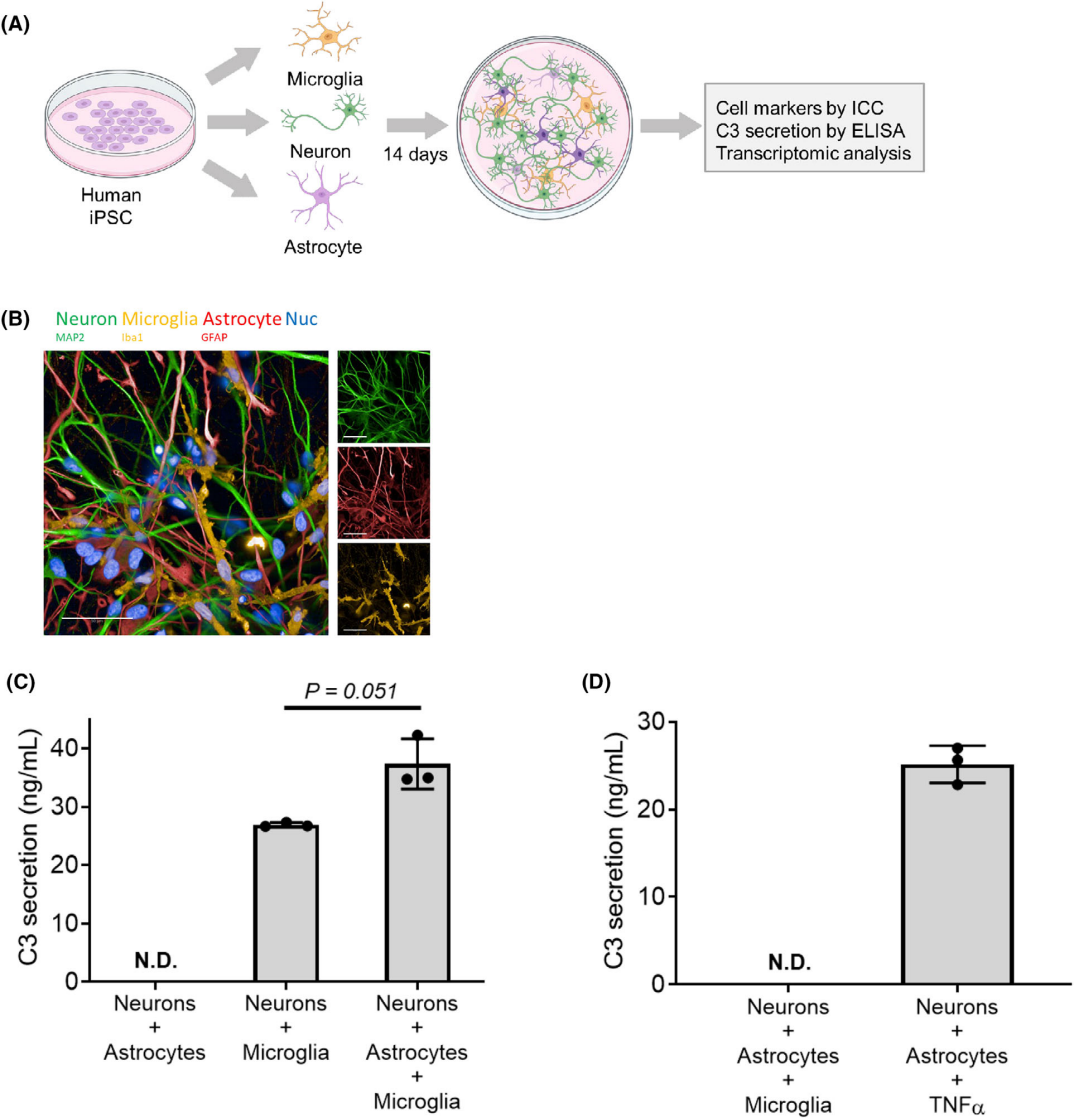
Human iPSC‐derived microglia cultured with iPSC‐derived astrocytes and neurons builds cellular crosstalk between microglia and astrocytes in an inflammatory loop. (A) Schematic of tri‐culture differentiation created by biorender. (B) Representative image of iPSC‐derived tri‐culture consisting of MAP2+ neurons (green), IBA1+ microglia (yellow) and GFAP+ astrocytes (red). Scale bar, 50 μm (*n* = 3 independent wells from one experiment). (C) Secreted C3 levels in the culture supernatant in neurons + astrocyte co‐culture, neuron + microglia co‐culture and tri‐culture by ELISA (mean ± SD, *n* = 3 independent wells from one experiment). Welch's *t*‐test, *P* = 0.051 (D) Secreted C3 levels in the culture supernatant in neurons + astrocyte co‐culture with TNF‐α treatment by ELISA (*n* = 3 independent wells from one experiment).

To better understand microglial function and cell–cell communication across culture systems, we established iPSC‐derived microglia cocultured with neurons and astrocytes, modified from multiple protocols described in previous reports [9, 10] (Fig. 1A). iPSC‐derived microglia were generated using a modified differentiation protocol expressing IBA1 [28]. iPSC‐derived neurons in the tri‐culture maintained their cortical identity and expressed MAP2, with commercially available iPSC‐derived iCell astrocytes expressing GFAP (Fig. 1B). All cell types formed a complex network within 2 weeks since triple culturing.

We further assessed the interplay among microglia, neurons, and astrocytes in our iPSC tri‐culture by measuring C3 secretion using ELISA (Fig. 1C,D). In the absence of any perturbation, C3 secretion was detected only when microglia were present, either in coculture with neurons or under tri‐culture conditions. There was a significant increase in C3 secretion in the tri‐culture compared to that in the neuron and microglia coculture (Fig. 1C). These results replicated previously published results, suggesting the impact of microglia and astrocyte interactions in tri‐culture regulating C3 production [9]. Additionally, we confirmed the presence of TNF‐α, a pro‐inflammatory cytokine that could lead to C3 secretion in neurons and astrocytes co‐culture without microglia (Fig. 1D). As TNF‐α can be secreted by microglia, it is possible that microglia may exert impact on C3 production via TNF‐α. Our findings are consistent with a previous report suggesting a feedback loop between microglia and astrocytes [9].

### Microglia transcriptional states reveal significant complexity in tri‐culture

**Fig. 2:**
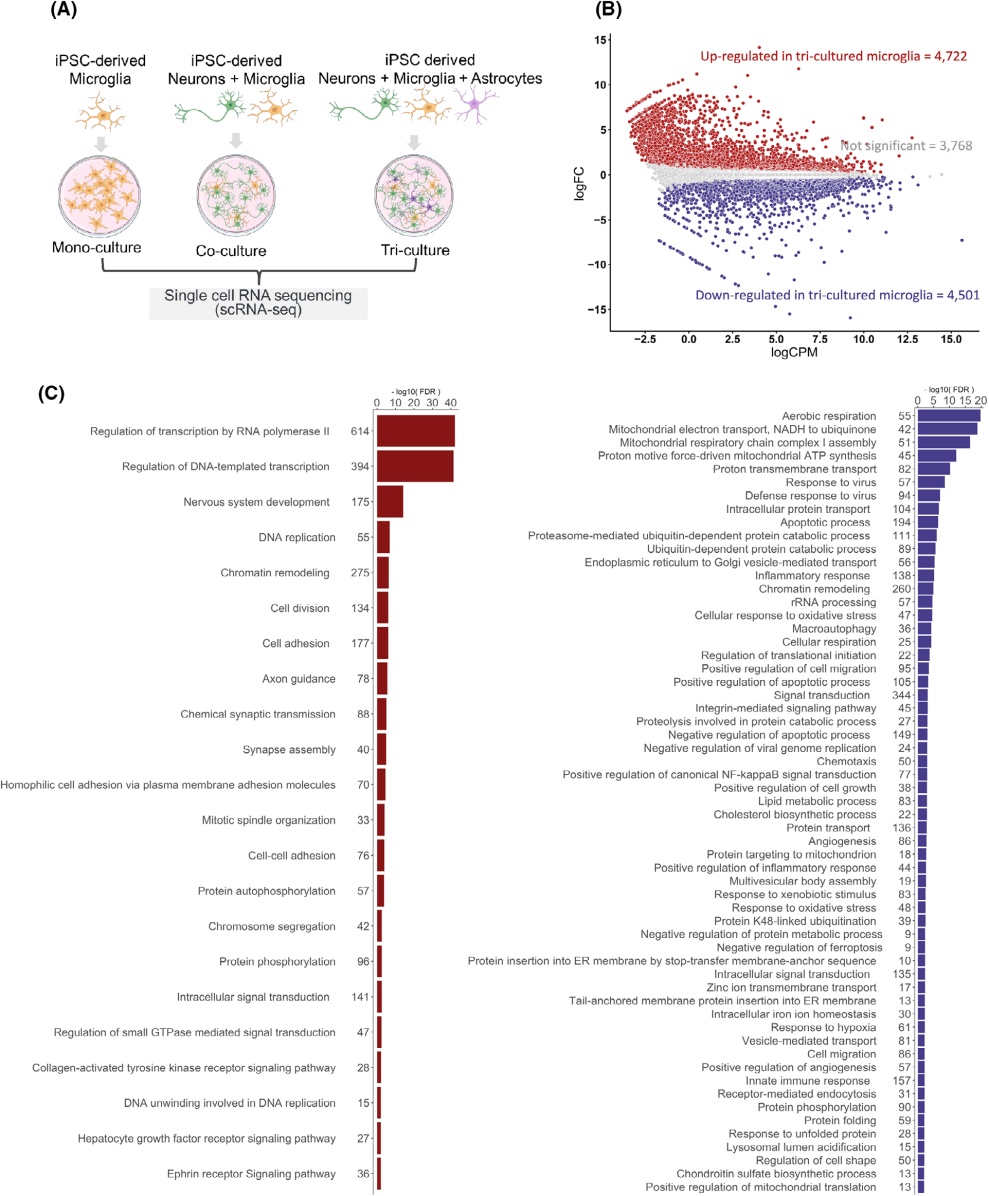
Transcriptomic feature comparison of monocultured and tri‐cultured microglia. (A) Schematic for the culture conditions subjected to scRNA‐seq created by biorender. (B) Comparison of gene expression levels between monocultured and tri‐cultured microglia. Significantly differentially expressed genes were identified by using edgeR, and the multiple comparison was adjusted by Benjamini–Hochberg (BH). Upregulated differentially expressed genes (DEGs) are highlighted in red and downregulated DEGs in blue (FDR < 0.05). (C) Enriched gene ontology biological processes for upregulated genes in tri‐cultured microglia (red brown) and in monocultured microglia (blue) Fisher's exact‐test was used, and *P*‐values in the test were adjusted by BH.

To further understand microglial state changes in the co‐culture of neurons and tri‐cultures of neurons and astrocytes, we performed scRNA‐seq (Fig. 2A). We compared the gene expression between monocultured and tri‐cultured microglia and identified 4722 upregulated and 4501 downregulated genes in tri‐cultured microglia (Fig. 2B). The upregulated genes in tri‐cultured microglia were associated with neuron‐related functions, including nervous system development, axon guidance, and endocytic functions involving homophilic cell adhesion via plasma membrane adhesion molecules (red bar plot in Fig. 2C). In contrast, the genes downregulated in the tri‐culture were enhanced for mitochondrial electron transport, response to viruses, and cellular response to oxidative stress (blue one in Fig. 2C).

**Fig. 3:**
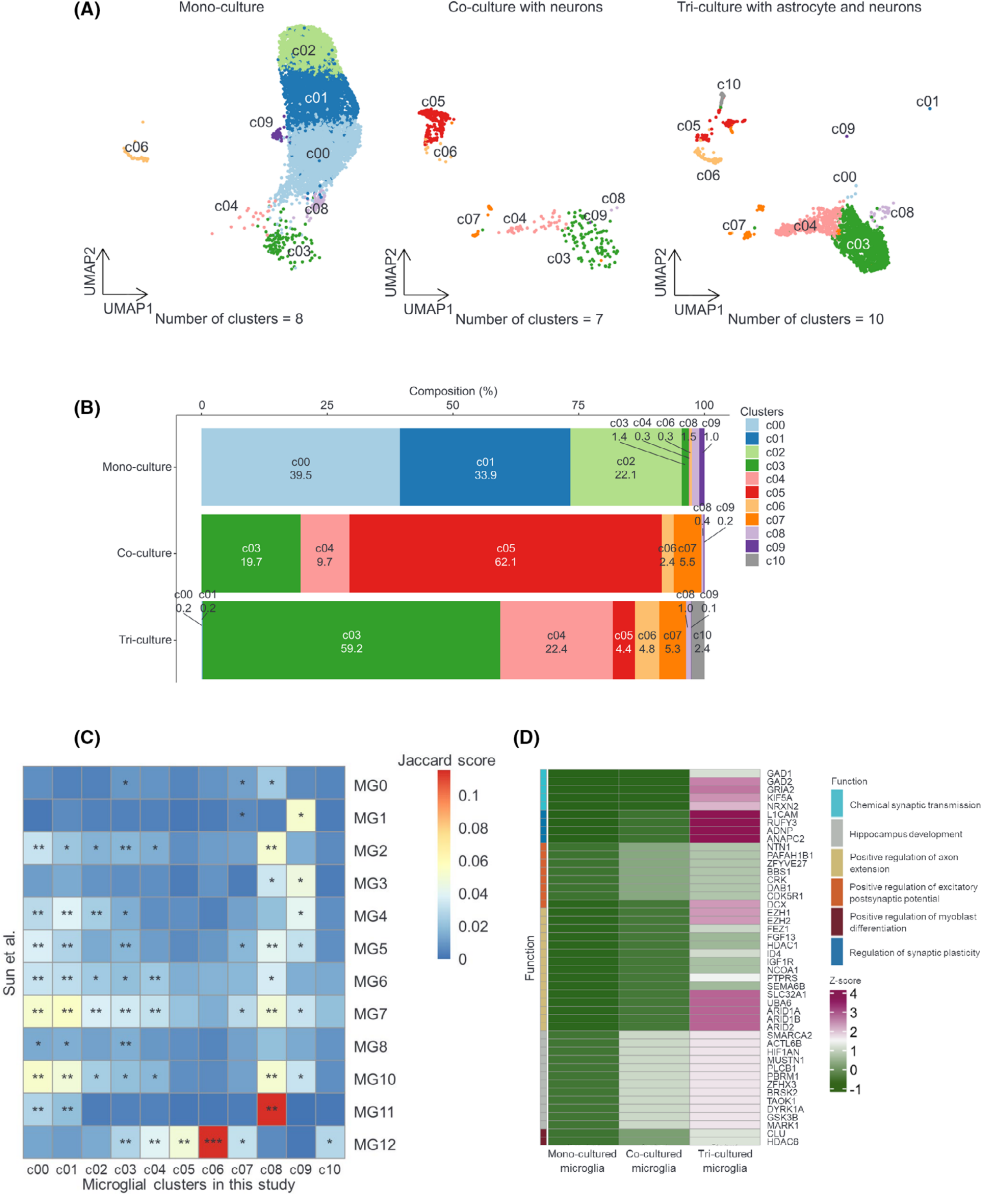
Microglia clustering in monoculture, co‐culture, and tri‐culture by scRNAseq. (A). UMAP representing microglial substates in the scRNA‐seq data from mono, co‐, and tri‐cultured microglia. (B) Cell fraction of each microglial cluster in the scRNA‐seq data. (C) Heatmap showing the significant overlap of marker genes in each microglial substate between Sun et al. and this study. Jaccard score represents the percentage of pairwise overlapping genes. Fisher's exact test was used to evaluate the significance (*P*‐value was adjusted by BH). (D) Expression levels of genes involved in neural functions. These functions were enriched in the microglial cluster c07 in this study.

To precisely characterize the microglial states, we defined clusters as distinct microglial states based on their transcriptomic signatures (Fig. 3A). Next, we compared these 10 microglial clusters with the *in vivo* microglial substrates described by Sun et al. [29]. Monoculture‐specific clusters (c00, c01, and c02; Fig. 3B) shared expression patterns with MG7 and MG10 (Fig. 3B) as defined by Sun et al. [29]. MG7 is enriched in glycolytic processes and switches from oxidative phosphorylation to glycolysis. A metabolic switch occurs when the microglia are exposed to inflammatory stimuli [30, 31]. MG10 has been annotated as inflammatory and shows high expression of cytokine receptors [29]. Clusters c03 and c04 comprised < 2% of the monocultured microglial cells, whereas these clusters accounted for approximately 80% of their tri‐cultured counterparts (Fig. 3B). c03 was enriched in endocytic recycling, trans‐Golgi network membranes, and regulation of macroautophagy (Fig. S1), suggesting that endocytosis could be activated in this cluster. A previous study has shown that interactions between microglia and injured motor neurons activate endocytosis [32], implying that interactions with neurons or astrocytes could contribute to the activation of endocytosis. Interestingly, c07 was found only in the co‐cultured and tri‐cultured microglia (Fig. 3A,B) and was enriched in neuron‐related functions, including chemical synaptic transmission, positive regulation of axon extension, and positive regulation of excitatory postsynaptic potential (Fig. S1). Next, we investigated whether the highly expressed genes in the microglial c07 cluster were modulated by interactions with astrocytes or neurons. We assessed the expression of neuronal function‐related genes that were enriched in c07 in monocultured, co‐cultured, and tri‐cultured microglia (Fig. 3D). All genes involved in these functions showed higher expression in tri‐cultured microglia than in monocultured microglia, suggesting that the expression of these genes could be modulated by the interaction of microglia with neurons or astrocytes [33, 34]. Genes involved in chemical synaptic transmission were also expressed in excitatory and inhibitory neurons, whereas excitatory postsynaptic potential‐related genes had the highest expression levels in astrocytes. Astrocytes play a role in synapse development through their interactions with synapses and modulate excitatory neurotransmission [35, 36]. Also, expression levels of genes involved in the positive regulation of axon extension were higher in inhibitory neurons than in excitatory ones. Inhibitory plasticity and remodeling could be useful for calibrating activity throughout the whole nervous system [35, 37], and inhibitory synapses are thought to be involved in regulating excitation via neural networks [38], which could lead to higher expression of these axon extension‐related genes.

### Microglia as a key player responding to iron and ferroptosis inducer

**Fig. 4:**
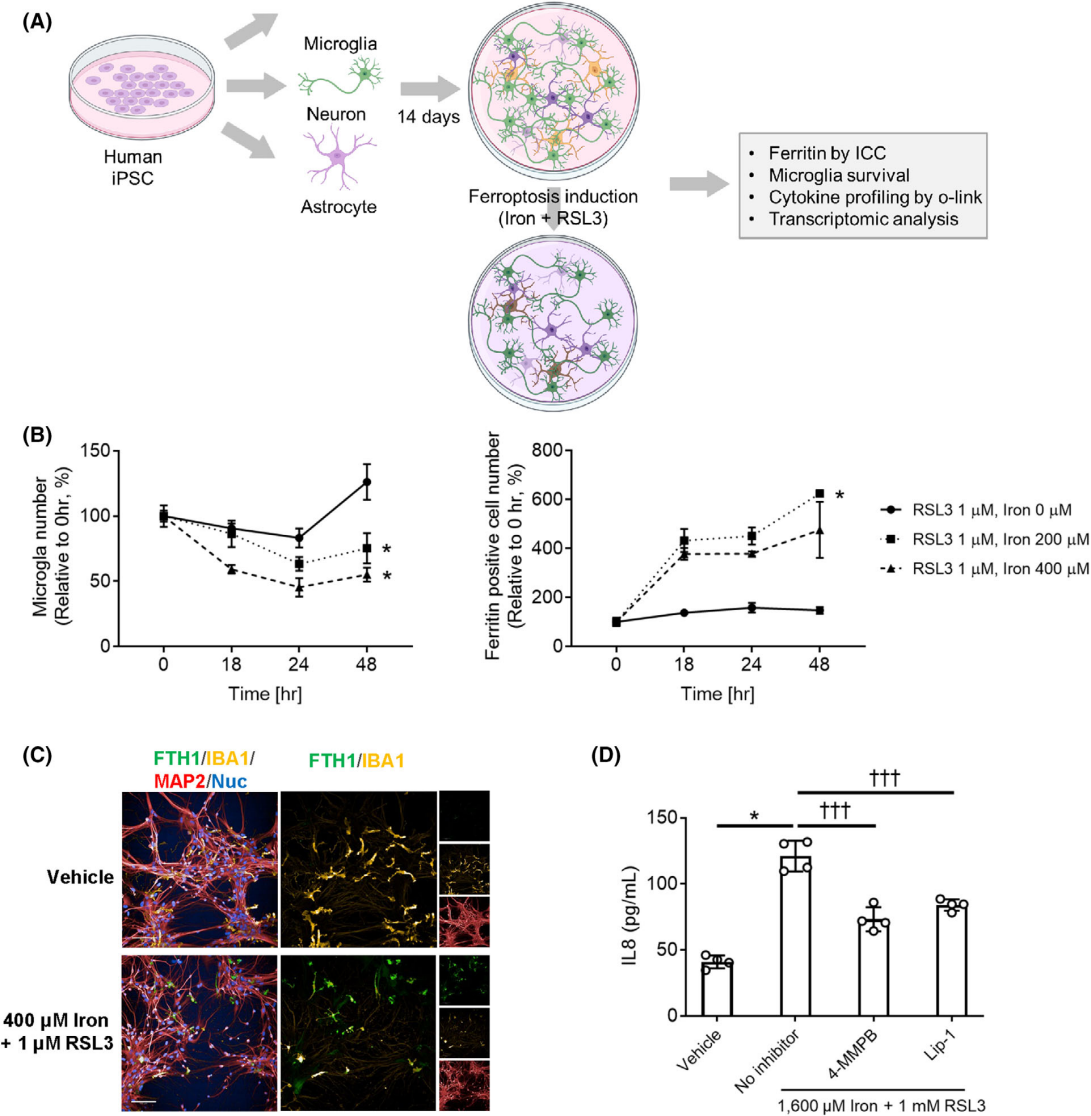
Ferroptosis induction causes microglia cell death and pro‐inflammatory activation in iPSC tri‐culture. (A). Schematic of ferroptosis induction in iPSC tri‐culture created by BioRender. (B). IBA1 positive microglia and FTH1 positive microglia cell numbers in tri‐culture with 200 or 400 μm iron +1 μm RSL3. The cell numbers were normalized to each 0 h post the treatment (mean ± SD, *n* = 3 independent wells from one of three independent experiments). All experiments show the same trend, one‐way ANOVA with Dunnett *P < 0.05. (C). Representative images of FTH1 (green) expression in IBA1+ microglia (yellow) in tri‐culture on day 1 with exposure to 400 μm iron +1 μm RSL3. Scale bar, 100 μm. (D). IL‐8 production in tri‐culture with iron + RSL3 with/without 10 μm lip‐1 or 10 μm benzothiazine (mean ± SD, *n* = 3 independent wells from one of three independent experiments). All experiments show the same trend; Student's *t*‐test, *P < 0.05, and one‐way ANOVA with Dunnett, ††† *P* < 0.0001.

To establish an *in vitro* model for the evaluation of iron‐induced effects, we evaluated the microglial responses to iron overload and ferroptosis inducers in the tri‐culture. Microglial cell survival, ferritin expression, cytokine production, and transcriptomic analyses were conducted in iPSC tri‐culture following iron and RSL3 treatments (Fig. 4A). As iron or RSL3 alone led to minimal cell death, as shown in a previous study [10], we evaluated cell survival under three iron concentrations with 1 μm RSL3. Microglia undergo cell death in tri‐culture with 200 μm iron and 1 μm RSL3, within 24–48 h of treatment. Greater cell death was identified with 400 μm iron and 1 μm RSL3 treatment, wherein after 48 h of treatment, the microglia were significantly decreased in number (Fig. 4B). While the astrocyte number didn't change over the treatment with 1600 μm iron and 1 μm RSL3 (Fig. S2). Ferritin heavy chain (FTH1) was co‐localized with IBA1, suggesting that ferroptosis was induced in the microglia (Fig. 4C). We also observed that IL‐8 secretion induced by the iron + RSL3 treatment was significantly inhibited by the antioxidants and ferroptosis inhibitors 4‐MMPB and lip‐1 (Fig. 4D).

**Fig. 5:**
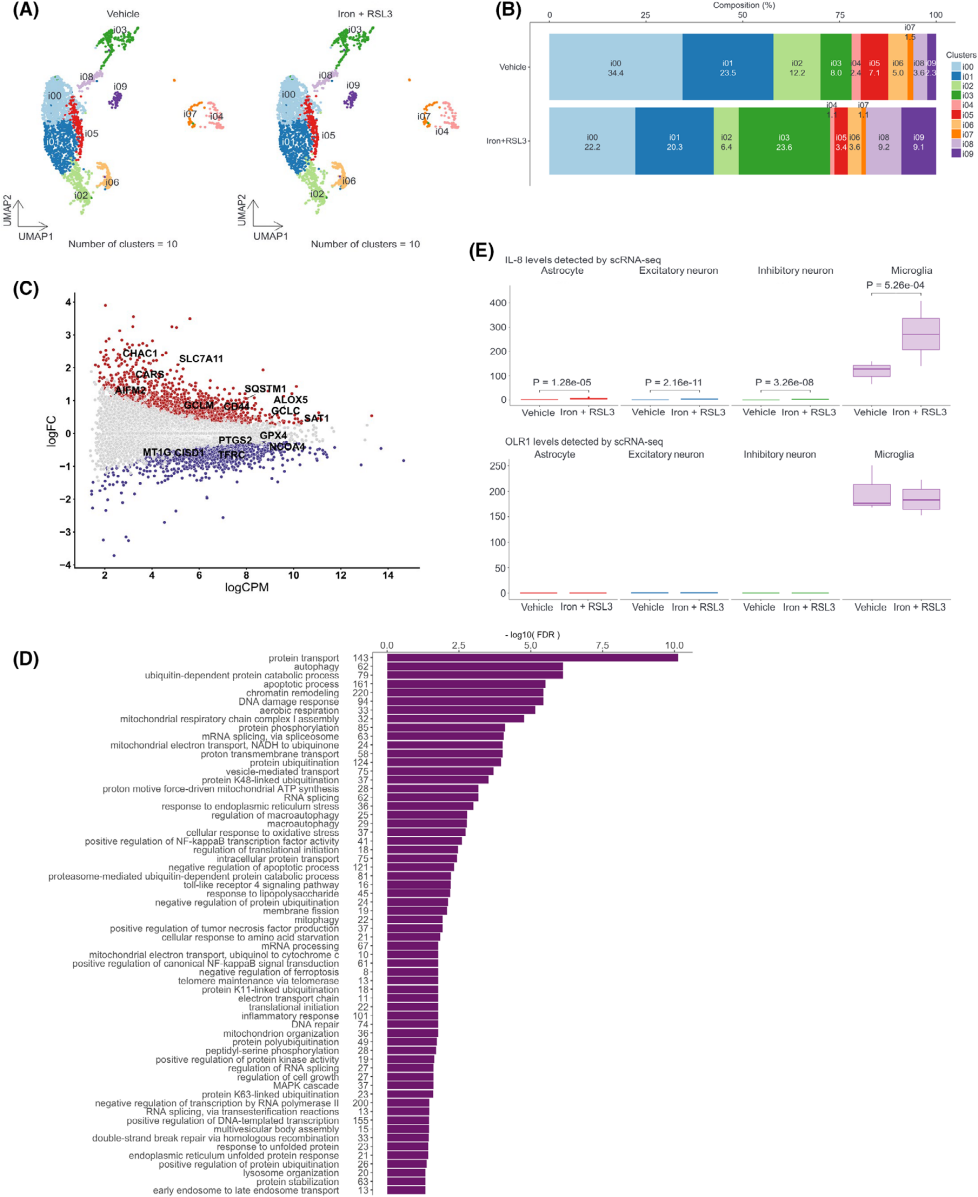
Microglia with ferroptosis induction causes a unique transcriptional response in iPSC tri‐culture. (A). UMAP of microglial clustering in the scRNA‐seq data from tri‐cultures exposed to vehicle or iron + RSL3. (B). Cell fraction distribution in microglial clusters from tri‐cultures. (C). Significantly differentially expressed genes in tri‐cultured microglia exposed to between vehicle and iron + RSL3 (FDR < 0.05). Pseudobulk gene expression levels were compared by using edgeR. Red dots represent upregulated genes in the iron + RSL3 treatment, and blue ones are upregulated in vehicle. (D). Functional annotation for significantly differentially expressed genes by the exposure to iron + RSL3. Fisher's exact test was used for gene ontology biological processes, and FDRs were calculated based on Benjamini and Hochberg. (E). IL‐8 and OLR1 expression levels in tri‐culture with iron + RSL3 treatment by pseudobulk scRNA‐seq analysis. Expression levels were compared by edgeR. Adjusted *P*‐values by BH were indicated in the graph.

To assess the inflammatory response for a longer period of time, we profiled inflammatory cytokines in the supernatant of tri‐cultures on days 1, 3, and 7 after iron + RSL3 treatment using Olink. Twenty‐eight out of the 45 cytokines were detected in the supernatant. Among these, IL‐8 and OLR1 levels changed significantly at all time points. This increase in IL‐8 levels was significantly reduced by 4‐MMPB treatment (Table 1), suggesting that IL‐8 may be regulated downstream of GPX4, a key enzyme involved in ferroptosis regulation. Reportedly, OLR1 mediates high glucose‐induced ferroptosis, which is linked to iron‐induced ferroptosis [39]. Regarding the mRNA levels, both IL‐8 and OLR1 were predominantly expressed in microglia at levels over 100‐fold higher than in other cell types (Fig. 5E). Therefore, microglia have been implicated as the main cell type producing cytokines that respond to iron‐induced ferroptosis.

**Table 1:** Secreted inflammatory proteins in tri‐cultures upon ion + RSL3 treatment (*n* = 3).

Assay	Fold change 1: Iron + RSL3 vs vehicle			Fold change 2: 4‐MMPB vs vehicle, with iron + RSL3		
	Day 1	Day 3	Day 7	Day 1	Day 3	Day 7
CXCL8/IL8	**2.63** *	**3.63** *	**3.04** *	**−1.58** *	**−1.47** *	**−1.8** *
IL6	**1.72** *	−1.08	−1.21	−1.37	−1.13	−1.32
MMP12	**1.56** *	1.19	**−1.32** *	−1.12	−1.15	1.09
OLR1	**1.32** *	**1.33** *	**1.37** *	−1.01	1.45	−1.00
HGF	**−2.36** *	−1.94	−2.58	1.15	1.28	−1.20
VEGFA	**−1.22** *	−1.17	**−1.51** *	1.04	1.14	1.14
TNFSF12	**−1.12** *	−1.09	**−2.25** *	−1.12	−1.37	−1.08
CCL8	1.36	2.26	**−3.06** *	−1.25	**−1.85** *	1.28
CCL4	1.28	1.22	**3.14** *	**−1.51** *	−1.36	**−1.50** *
IL18	1.27	1.49	−1.08	−1.06	−1.08	1.01
CCL3	1.26	1.15	**1.58** *	−1.41	−1.20	**−1.48** *
OSM	1.23	1.06	**−1.31** *	−1.11	−1.26	1.12
IL1B	−1.01	**1.35** *	1.02	1.08	**−1.51** *	−1.42
IFNG	−1.08	1.05	−1.02	1.01	−1.07	−1.01
FLT3LG	−1.09	1.27	1.07	1.12	−1.16	−1.33
TNF	−1.12	1.03	−1.18	1.10	**−1.17**	−1.08
IL15	−1.15	1.27	−1.23	1.12	1.05	−1.26
TGFA	−1.16	1.20	−1.71	−1.07	**−1.94** *	−1.08
MMP1	−1.30	1.17	**−2.82** *	**−1.47** *	**−1.99** *	−1.03
CXCL12	−1.34	1.09	−1.31	1.37	−1.15	**−1.81** *
EGF	−1.35	−1.00	−1.22	1.19	−1.12	1.16
CSF2	−1.36	1.20	−1.57	1.12	−1.10	−1.04
IL2	−1.39	−1.00	−1.37	1.09	−1.15	−1.28
CCL11	−1.47	**2.88** *	−1.17	−1.32	**−2.57** *	−1.13
IL33	−1.56	1.33	−1.31	1.56	−1.21	**−1.83** *
CSF3	−1.78	1.10	−2.24	1.28	−1.40	−1.21
IL7	−1.87	−1.08	−1.94	1.25	**−1.47** *	−1.30
IL17A	−1.96	−1.10	−1.71	1.30	1.63	**−1.47** *

Significance of bold values meets *p*‐value < 0.05 by Welch's *t*‐test.

* *P* < 0.05 (Welch's *t*‐test).

### Ferroptosis induction changes the key clusters of microglia in iPSC tri‐culture

To better understand and characterize the microglial features in this tri‐culture, we applied iron + RSL3 perturbation and performed scRNA‐seq analysis. The neuron‐to‐astrocyte‐to‐microglia ratios are shown in Table S1.

To assess microglial characteristics in more detail, microglial populations were clustered into 10 subclusters based on their expression patterns (Fig. 5A,B), and biological pathways for each subcluster were annotated based on the enrichment of gene expression signatures (Fig. S3). The proportion of subclusters indicated that the i00 and i02 clusters drastically decreased. These clusters have been implicated in the endocytic and kinase signaling pathways. In contrast, the proportions of clusters i03, i08, and i09, which are linked with neuronal function regulation (e.g., presynaptic active zone and SNARE complex), increased upon iron + RSL3 treatment (Figs 5B and S3). To investigate the whole microglial response to ion+RSL3 treatment, we performed a pseudobulk analysis to understand the overall gene expression changes. We identified 1770 upregulated and 1824 downregulated genes in response to iron + RSL3 treatment (Fig. 5C). Some of these genes are ferroptosis‐related, a finding consistent with that of a previous study of microglial ferroptosis in an iPSC‐multicellular co‐culture [10]. These differentially expressed genes were enriched in the negative regulation of ferroptosis, autophagy, response to endoplasmic reticulum stress, and cellular response to oxidative stress (Fig. 5D), suggesting that the activities of ferroptosis‐related pathways were altered in response to iron overload.

## Discussion

We successfully developed a human iPSC‐derived neuron–astrocyte–microglia tri‐culture model that offers a valuable platform for studying microglial dynamics and their interactions with neurons and astrocytes. By integrating cellular phenotype characterization with scRNA‐seq analysis, this model provided significant insights into the cellular and molecular mechanisms underlying glia–neuron interactions.

Our scRNA‐seq analysis focused on microglial gene expression as distinct transcriptional signatures, while preserving core microglial identity, which may provide clues regarding functional variations. In monoculture, microglia displayed a transcriptional profile indicative of heightened inflammatory responses, including elevated expression of cytokines such as *IL1B*. In contrast, in the tri‐culture system, interactions between neurons and astrocytes reshaped microglial gene expression and upregulated pathways related to nervous system development, axon guidance, and endocytosis. We used different media for monoculture and tri‐culture conditions. This was because monocultured microglia could not survive in the same medium as tri‐culture, suggesting that the possibility that those transcriptional changes may be attributable to specific components. However, our observations align with recent findings highlighting how microglia behave differently in monoculture versus tri‐culture systems, influenced by cross talk with neurons and astrocytes. Two recent studies using similar tri‐culture approaches applied scRNA‐seq and C3 ELISA, revealing how microglial interactions with astrocytes and neurons alter intercellular communication pathways and functional states [40, 41]. These findings further support the utility and necessity of tri‐culture models reflecting, at least partially, the complexity of the CNS environment for uncovering mechanisms of cellular interplay in healthy and neurodegenerative conditions.

Dysfunction of microglia is an important pathogenic mechanism for Alzheimer's disease. An important clue about potential triggers of microglial degeneration was provided by observations showing that many dystrophic microglia are positive for the iron storage protein, ferritin, and that such iron‐laden microglia are prevalent in AD brain [11, 42]. Kenkhuis *et al*. [43] identified that these morphologically dystrophic microglia exhibit high expression of the iron storage protein ferritin light chain (FTL) together with increased IBA1 expression and decreased TMEM119 and P2RY12 expression, particularly in patients with high Aβ load and Tau load. Combining the findings that the appearance of dystrophic microglia often precedes tau‐pathology and ferritin‐positive microglia are observed near neuritic plaques in AD brains [44, 45], it is reasonable to hypothesize that ferritin‐expressing microglia maybe a first line of defense against elevated iron levels, which increase with age and are also elevated in the AD brains. A neuron, astrocytes, and microglia tri‐culture system will be useful to assess how iron‐laden microglia impact neuronal function and health.

Our study revealed that microglia in the iPSC‐derived tri‐culture model undergo ferroptosis upon exposure to iron and the GPX4 inhibitor RSL3, as evidenced by the increased expression of ferroptosis‐related genes and markers, including FTH1. This is consistent with previously published studies in which microglia susceptibility to ferroptosis can be induced in tri‐culture conditions [10]. By examining enriched genes and using KEGG pathway analysis, Ryan *et al*. reported three clusters of microglia that are enriched with ferroptosis‐associated signatures, proliferative or homeostatic gene expression signatures, respectively. The gene expression profile of the ferroptosis‐associated subcluster (FAS) of microglia correlates with ferroptosis and lipid pathways, as well as p53 signaling, autophagy, and endoplasmic reticulum (ER) stress. In contrast to their findings, we observed changes in the proportion of microglia subclusters with enriched gene expressions related to endocytic pathways and neuronal regulation pathways following iron overload exposure. To understand the difference in the transcriptomic profile observed in those previous studies, we need to further evaluate experimental conditions and compare gene expression analysis algorithms. Nevertheless, the observed microglial cell death and ferroptosis marker expression allow us to further characterize the relevance of our tri‐culture model in disease pathophysiology. Gene expression analysis to reveal changes in pathways associated with ferroptosis, endoplasmic reticulum stress, and cellular oxidative responses will be needed before applying such a tri‐culture model as a valuable tool to study neurodegeneration.

Further studies are necessary to delineate how microglia influence neuronal health and disease pathology. This is particularly relevant as GWAS and functional genomics studies of AD illustrate the cell type‐specific impact of microglia on genetic risk variants [46, 47]. Characterizing the effects of genetic variations and disease‐related perturbations on microglial function in this model, combined with investigating the role of other CNS cell types and their interactions with microglia within such a tri‐culture system, will advance our understanding of neurodegenerative processes and their therapeutic targets.

In conclusion, the iPSC‐derived tri‐culture system represents a robust and physiologically relevant platform for exploring microglial biology and cellular CNS interactions. The findings from this study not only enhance our understanding of microglial function but also lay the foundation for developing innovative therapeutic strategies to combat neurodegenerative diseases.

## Conflicts of interest

All the authors are employees of Takeda Pharmaceutical Co., Ltd.

## Author contributions

HLL, MY, YL contributed to conceptualization; HLL, HO, SS, MY, AO contributed to investigation; HLL, HO, SS, MY, AO contributed to formal analysis and visualization; MF, YL contributed to supervision; HLL, HO, SS, MY contributed to writing and original draft; HLL, MY, YL contributed to writing and review and editing.

## Supporting information


**Table S1.** Cell fraction of each cell type in iPSC‐derived tri‐cultures in different condition by scRNA‐seq.
**Fig. S1.** Enriched gene ontology biological processes in microglial clusters.
**Fig. S2.** Ferroptosis induction does not affect the number of astrocytes in the iPSC tri‐culture.
**Fig. S3.** Enriched gene ontology biological processes in microglial clusters upon ion + RSL3 treatment.

## Data Availability

The data that support the findings of this study are available from the corresponding author [yan.ling@takeda.com] upon reasonable request.

## References

[1] Sierra A , Abiega O , Shahraz A and Neumann H (2013) Janus‐faced microglia: beneficial and detrimental consequences of microglial phagocytosis. Front Cell Neurosci 7, 6. 23386811 10.3389/fncel.2013.00006 PMC3558702

[2] Crotti A and Ransohoff RM (2016) Microglial physiology and pathophysiology: insights from genome‐wide transcriptional profiling. Immunity 44, 505–515. 26982357 10.1016/j.immuni.2016.02.013

[3] Bohlen CJ , Friedman BA , Dejanovic B and Sheng M (2019) Microglia in brain development, homeostasis, and neurodegeneration. Annu Rev Genet 53, 263–288. 31518519 10.1146/annurev-genet-112618-043515

[4] Ben Fredj N , Chaabane A , Chadly Z , Ben Fadhel N , Boughattas NA and Aouam K (2014) Albendazole‐induced associated acute hepatitis and bicytopenia. Scand J Infect Dis 46, 149–151. 24423162 10.3109/00365548.2013.835068

[5] Clites BL and Pierce JT (2017) Identifying cellular and molecular mechanisms for magnetosensation. Annu Rev Neurosci 40, 231–250. 28772099 10.1146/annurev-neuro-072116-031312 PMC5588146

[6] Sabarinath VP , Hazarey PV , Sharma N and Jain S (2009) Precise, simultaneous bracket and miniscrew placement. J Clin Orthod 43, 513–514. 19904043

[7] Wilton DK , Dissing‐Olesen L and Stevens B (2019) Neuron‐glia signaling in synapse elimination. Annu Rev Neurosci 42, 107–127. 31283900 10.1146/annurev-neuro-070918-050306

[8] Kriegmair MC , Speck T , Schneider AW , Volkmer B and Michel MS (2021) Urological care in practices and clinics during the corona virus pandemic in Germany. Urologe A 60, 318–330. 33559694 10.1007/s00120-021-01458-z PMC7871316

[9] Guttikonda SR , Sikkema L , Tchieu J , Saurat N , Walsh RM , Harschnitz O , Ciceri G , Sneeboer M , Mazutis L , Setty M *et al*. (2021) Fully defined human pluripotent stem cell‐derived microglia and tri‐culture system model C3 production in Alzheimer's disease. Nat Neurosci 24, 343–354. 33558694 10.1038/s41593-020-00796-z PMC8382543

[10] Ryan SK , Zelic M , Han Y , Teeple E , Chen L , Sadeghi M , Shankara S , Guo L , Li C , Pontarelli F *et al*. (2023) Microglia ferroptosis is regulated by SEC24B and contributes to neurodegeneration. Nat Neurosci 26, 12–26. 36536241 10.1038/s41593-022-01221-3 PMC9829540

[11] Lopes KO , Sparks DL and Streit WJ (2008) Microglial dystrophy in the aged and Alzheimer's disease brain is associated with ferritin immunoreactivity. GLIA 56, 1048–1060. 18442088 10.1002/glia.20678

[12] Streit WJ , Sammons NW , Kuhns AJ and Sparks DL (2004) Dystrophic microglia in the aging human brain. GLIA 45, 208–212. 14730714 10.1002/glia.10319

[13] Streit WJ , Khoshbouei H and Bechmann I (2021) The role of microglia in sporadic Alzheimer's disease. J Alzheimer's Dis 79, 961–968. 33361603 10.3233/JAD-201248

[14] Adeniyi PA , Gong X , MacGregor E , Degener‐O'Brien K , McClendon E , Garcia M , Romero O , Russell J , Srivastava T , Miller J *et al*. (2023) Ferroptosis of microglia in aging human White matter injury. Ann Neurol 94, 1048–1066. 37605362 10.1002/ana.26770 PMC10840747

[15] Shi Y , Kirwan P and Livesey FJ (2012) Directed differentiation of human pluripotent stem cells to cerebral cortex neurons and neural networks. Nat Protoc 7, 1836–1846. 22976355 10.1038/nprot.2012.116

[16] Shi Y , Kirwan P , Smith J , Robinson HP and Livesey FJ (2012) Human cerebral cortex development from pluripotent stem cells to functional excitatory synapses. Nat Neurosci 15, 477–486. 22306606 10.1038/nn.3041 PMC3882590

[17] Okuzono Y , Sakuma H , Miyakawa S , Ifuku M , Lee J , Das D , Banerjee A , Zhao Y , Yamamoto K , Ando T *et al*. (2021) Reduced TREM2 activation in microglia of patients with Alzheimer's disease. FEBS Open Bio 11, 3063–3080. 10.1002/2211-5463.13300 PMC8564098 34523252

[18] Zheng GX , Terry JM , Belgrader P , Ryvkin P , Bent ZW , Wilson R , Ziraldo SB , Wheeler TD , McDermott GP , Zhu J *et al*. (2017) Massively parallel digital transcriptional profiling of single cells. Nat Commun 8, 14049. 28091601 10.1038/ncomms14049 PMC5241818

[19] Wolf FA , Angerer P and Theis FJ (2018) SCANPY: large‐scale single‐cell gene expression data analysis. Genome Biol 19, 15. 29409532 10.1186/s13059-017-1382-0 PMC5802054

[20] Mathys H , Peng Z , Boix CA , Victor MB , Leary N , Babu S , Abdelhady G , Jiang X , Ng AP , Ghafari K *et al*. (2023) Single‐cell atlas reveals correlates of high cognitive function, dementia, and resilience to Alzheimer's disease pathology. Cell 186, 4365–4385 e27. 37774677 10.1016/j.cell.2023.08.039 PMC10601493

[21] Wolock SL , Lopez R and Klein AM (2019) Scrublet: computational identification of cell doublets in single‐cell transcriptomic data. Cell Syst 8, 281–291 e9. 30954476 10.1016/j.cels.2018.11.005 PMC6625319

[22] Hu C , Li T , Xu Y , Zhang X , Li F , Bai J , Chen J , Jiang W , Yang K , Ou Q *et al*. (2023) CellMarker 2.0: an updated database of manually curated cell markers in human/mouse and web tools based on scRNA‐seq data. Nucleic Acids Res 51, D870–D876. 36300619 10.1093/nar/gkac947 PMC9825416

[23] Hao Y , Stuart T , Kowalski MH , Choudhary S , Hoffman P , Hartman A , Srivastava A , Molla G , Madad S , Fernandez‐Granda C *et al*. (2024) Dictionary learning for integrative, multimodal and scalable single‐cell analysis. Nat Biotechnol 42, 293–304. 37231261 10.1038/s41587-023-01767-y PMC10928517

[24] Korsunsky I , Millard N , Fan J , Slowikowski K , Zhang F , Wei K , Baglaenko Y , Brenner M , Loh PR and Raychaudhuri S (2019) Fast, sensitive and accurate integration of single‐cell data with harmony. Nat Methods 16, 1289–1296. 31740819 10.1038/s41592-019-0619-0 PMC6884693

[25] Sherman BT , Panzade G , Imamichi T and Chang W (2024) DAVID ortholog: an integrative tool to enhance functional analysis through orthologs. Bioinformatics 40, btae615. 39412445 10.1093/bioinformatics/btae615 PMC11520416

[26] Traag VA , Waltman L and van Eck NJ (2019) From Louvain to Leiden: guaranteeing well‐connected communities. Sci Rep 9, 5233. 30914743 10.1038/s41598-019-41695-z PMC6435756

[27] Robinson MD , McCarthy DJ and Smyth GK (2010) edgeR: a Bioconductor package for differential expression analysis of digital gene expression data. Bioinformatics 26, 139–140. 19910308 10.1093/bioinformatics/btp616 PMC2796818

[28] Abud EM , Ramirez RN , Martinez ES , Healy LM , Nguyen CHH , Newman SA , Yeromin AV , Scarfone VM , Marsh SE , Fimbres C *et al*. (2017) iPSC‐derived human microglia‐like cells to study neurological diseases. Neuron 94, 278–293 e9. 28426964 10.1016/j.neuron.2017.03.042 PMC5482419

[29] Sun N , Victor MB , Park YP , Xiong X , Scannail AN , Leary N , Prosper S , Viswanathan S , Luna X , Boix CA *et al*. (2023) Human microglial state dynamics in Alzheimer's disease progression. Cell 186, 4386–4403 e29. 37774678 10.1016/j.cell.2023.08.037 PMC10644954

[30] Lauro C and Limatola C (2020) Metabolic reprograming of microglia in the regulation of the innate inflammatory response. Front Immunol 11, 493. 32265936 10.3389/fimmu.2020.00493 PMC7099404

[31] Pan RY , He L , Zhang J , Liu X , Liao Y , Gao J , Liao Y , Yan Y , Li Q , Zhou X *et al*. (2022) Positive feedback regulation of microglial glucose metabolism by histone H4 lysine 12 lactylation in Alzheimer's disease. Cell Metab 34, 634–648. 35303422 10.1016/j.cmet.2022.02.013

[32] Salvany S , Casanovas A , Piedrafita L , Tarabal O , Hernandez S , Caldero J and Esquerda JE (2021) Microglial recruitment and mechanisms involved in the disruption of afferent synaptic terminals on spinal cord motor neurons after acute peripheral nerve injury. GLIA 69, 1216–1240. 33386754 10.1002/glia.23959 PMC7986680

[33] Wang Y , Fu AKY and Ip NY (2022) Instructive roles of astrocytes in hippocampal synaptic plasticity: neuronal activity‐dependent regulatory mechanisms. FEBS J 289, 2202–2218. 33864430 10.1111/febs.15878 PMC9290076

[34] Schober AL , Wicki‐Stordeur LE , Murai KK and Swayne LA (2022) Foundations and implications of astrocyte heterogeneity during brain development and disease. Trends Neurosci 45, 692–703. 35879116 10.1016/j.tins.2022.06.009

[35] Allen NJ and Eroglu C (2017) Cell biology of astrocyte‐synapse interactions. Neuron 96, 697–708. 29096081 10.1016/j.neuron.2017.09.056 PMC5687890

[36] Adermark L , Lagstrom O , Loften A , Licheri V , Havenang A , Loi EA , Stomberg R , Soderpalm B , Domi A and Ericson M (2022) Astrocytes modulate extracellular neurotransmitter levels and excitatory neurotransmission in dorsolateral striatum via dopamine D2 receptor signaling. Neuropsychopharmacology 47, 1493–1502. 34811469 10.1038/s41386-021-01232-x PMC9206030

[37] Froemke RC (2015) Plasticity of cortical excitatory‐inhibitory balance. Annu Rev Neurosci 38, 195–219. 25897875 10.1146/annurev-neuro-071714-034002 PMC4652600

[38] Fishell G and Rudy B (2011) Mechanisms of inhibition within the telencephalon: “where the wild things are”. Annu Rev Neurosci 34, 535–567. 21469958 10.1146/annurev-neuro-061010-113717 PMC3556485

[39] Zhang H , Chang X , Liu X , Zhang B , Wang R , Wang Y , Dai S , Yao T and Zhang Q (2025) Silencing of LOX‐1 attenuates high glucose‐induced ferroptosis in THVECs via the HIF‐1alpha/SLC7A11 signaling pathway. Exp Cell Res 446, 114451. 40015503 10.1016/j.yexcr.2025.114451

[40] Lish AM , Ashour N , Pearse RV 2nd , Galle PC , Orme GA , Heuer SE , Benoit CR , Alexander KD , Grogan EFL , Terzioglu G *et al*. (2025) Astrocyte induction of disease‐associated microglia is suppressed by acute exposure to fAD neurons in human iPSC triple cultures. Cell Rep 44, 115777. 40471789 10.1016/j.celrep.2025.115777 PMC12282607

[41] Lish AM , Grogan EFL , Benoit CR , Pearse RV 2nd , Heuer SE , Luquez T , Orme GA , Galle PC , Milinkeviciute G , Green KN *et al*. (2025) CLU alleviates Alzheimer's disease‐relevant processes by modulating astrocyte reactivity and microglia‐dependent synaptic density. Neuron 113, 1925–1946. 40311610 10.1016/j.neuron.2025.03.034 PMC12181066

[42] Simmons DA , Casale M , Alcon B , Pham N , Narayan N and Lynch G (2007) Ferritin accumulation in dystrophic microglia is an early event in the development of Huntington's disease. GLIA 55, 1074–1084. 17551926 10.1002/glia.20526

[43] Kenkhuis B , Somarakis A , de Haan L , Dzyubachyk O , Me IJ , de Miranda N , Lelieveldt BPF , Dijkstra J , van Roon‐Mom WMC , Hollt T *et al*. (2021) Iron loading is a prominent feature of activated microglia in Alzheimer's disease patients. Acta Neuropathol Commun 9, 27. 33597025 10.1186/s40478-021-01126-5 PMC7887813

[44] Streit WJ , Braak H , Xue QS and Bechmann I (2009) Dystrophic (senescent) rather than activated microglial cells are associated with tau pathology and likely precede neurodegeneration in Alzheimer's disease. Acta Neuropathol 118, 475–485. 19513731 10.1007/s00401-009-0556-6 PMC2737117

[45] Mayr E , Rotter J , Kuhrt H , Winter K , Stassart RM , Streit WJ and Bechmann I (2024) Detection of molecular markers of ferroptosis in human Alzheimer's disease brains. J Alzheimer's Dis 102, 1133–1154. 39529255 10.1177/13872877241296563

[46] Fujita M , Gao Z , Zeng L , McCabe C , White CC , Ng B , Green GS , Rozenblatt‐Rosen O , Phillips D , Amir‐Zilberstein L *et al*. (2024) Cell subtype‐specific effects of genetic variation in the Alzheimer's disease brain. Nat Genet 56, 605–614. 38514782 10.1038/s41588-024-01685-y PMC12288883

[47] Romero‐Molina C , Garretti F , Andrews SJ , Marcora E and Goate AM (2022) Microglial efferocytosis: diving into the Alzheimer's disease gene pool. Neuron 110, 3513–3533. 36327897 10.1016/j.neuron.2022.10.015 PMC13175419

