# Supplementary material for: Microglial dynamics and ferroptosis induction in human iPSC‐derived neuron–astrocyte–microglia tri‐cultures
Source: FEBS Open Bio. 2026 Jan 14;16(6):1060–73. doi: 10.1002/2211-5463.70182 (PMC13238601; doi:10.1002/2211-5463.70182)

Microglial dynamics and ferroptosis induction in human iPSC-derived neuron–astrocyte–microglia tri-cultures

Hongmei Lisa Li, Hiroko Ohmiya, Sou Sakamoto, Masato Yugami, Akiko Oki, Makoto Furusawa, Yan Ling*

Neuroscience Translational Medicine, Neuroscience Drug Discovery Unit, Research, Takeda Pharmaceutical Company, Fujisawa, Kanagawa, 2518555, Japan

Supplementary Table 1. Cell fraction of each cell type in iPSC derived tri-cultures in different condition by scRNA-seq.

Supplementary Figure 1. Enriched gene ontology biological processes in microglial clusters.

Supplementary Figure 2. Ferroptosis induction does not affect the number of astrocytes in the iPSC tri-culture.

Supplementary Figure 3. Enriched gene ontology biological processes in microglial clusters upon ion + RSL3 treatment.

Supplementary Table 1

Cell type	Composition (%)	
	Vehicle	Iron + RSL3
Microglia	10.2	7.2
Neuron	73.2	86.0
Astrocyte	16.6	6.8
Total	100.0	100.0

Supplementary Fig. 1

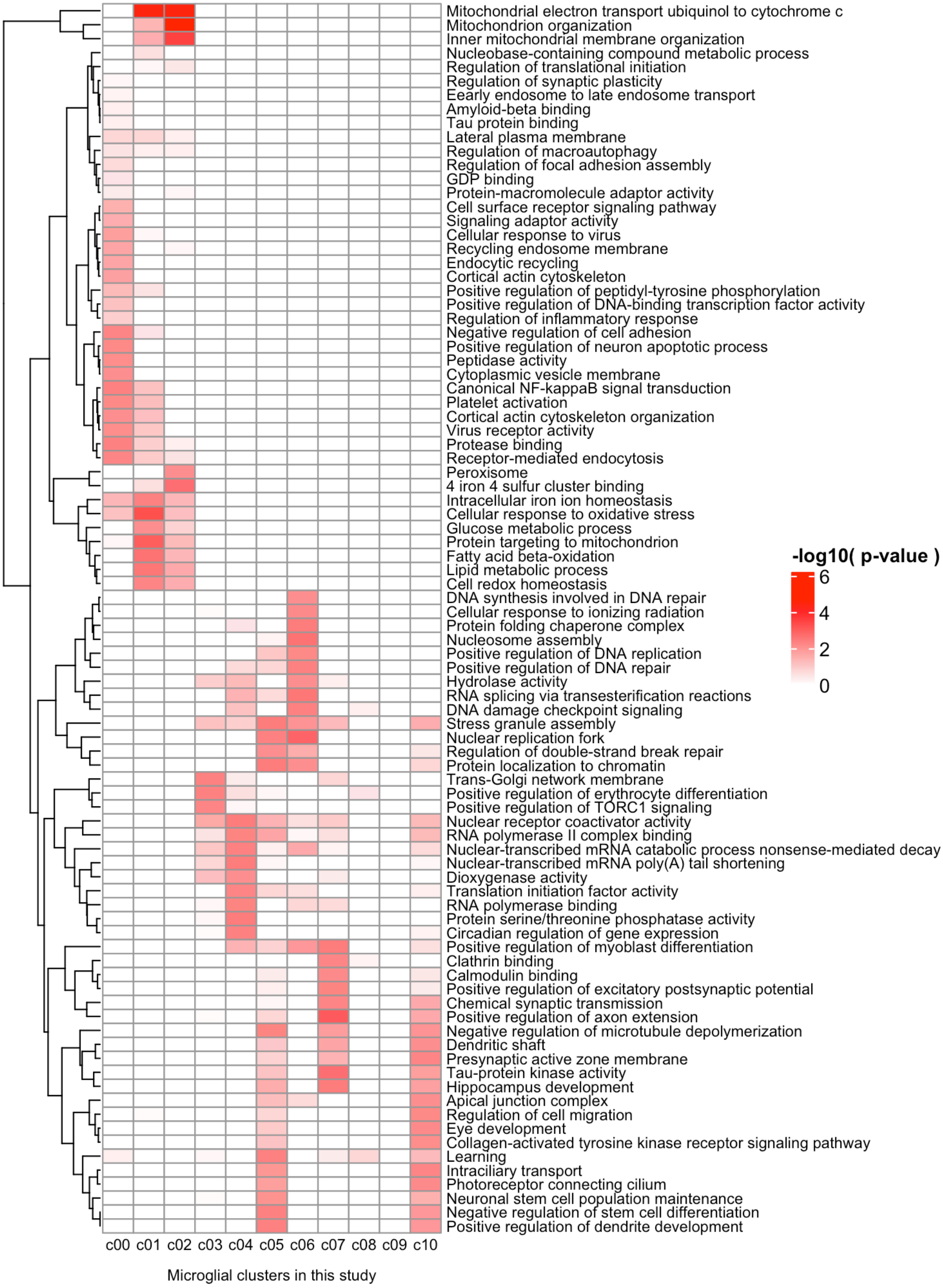

Supplementary Fig. 2

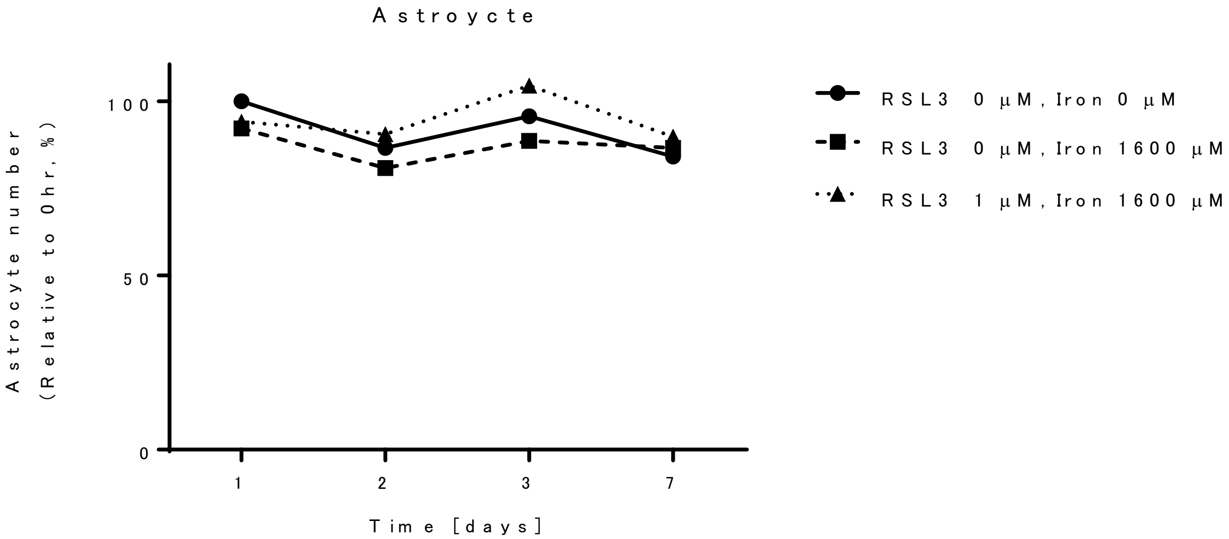

GFAP positive Astrocyte cell numbers in tri-culture with 1600 μM iron + 1 μM RSL3. The cell numbers were normalized to each 0 h post the treatment. n = 1

Supplementary Fig. 3

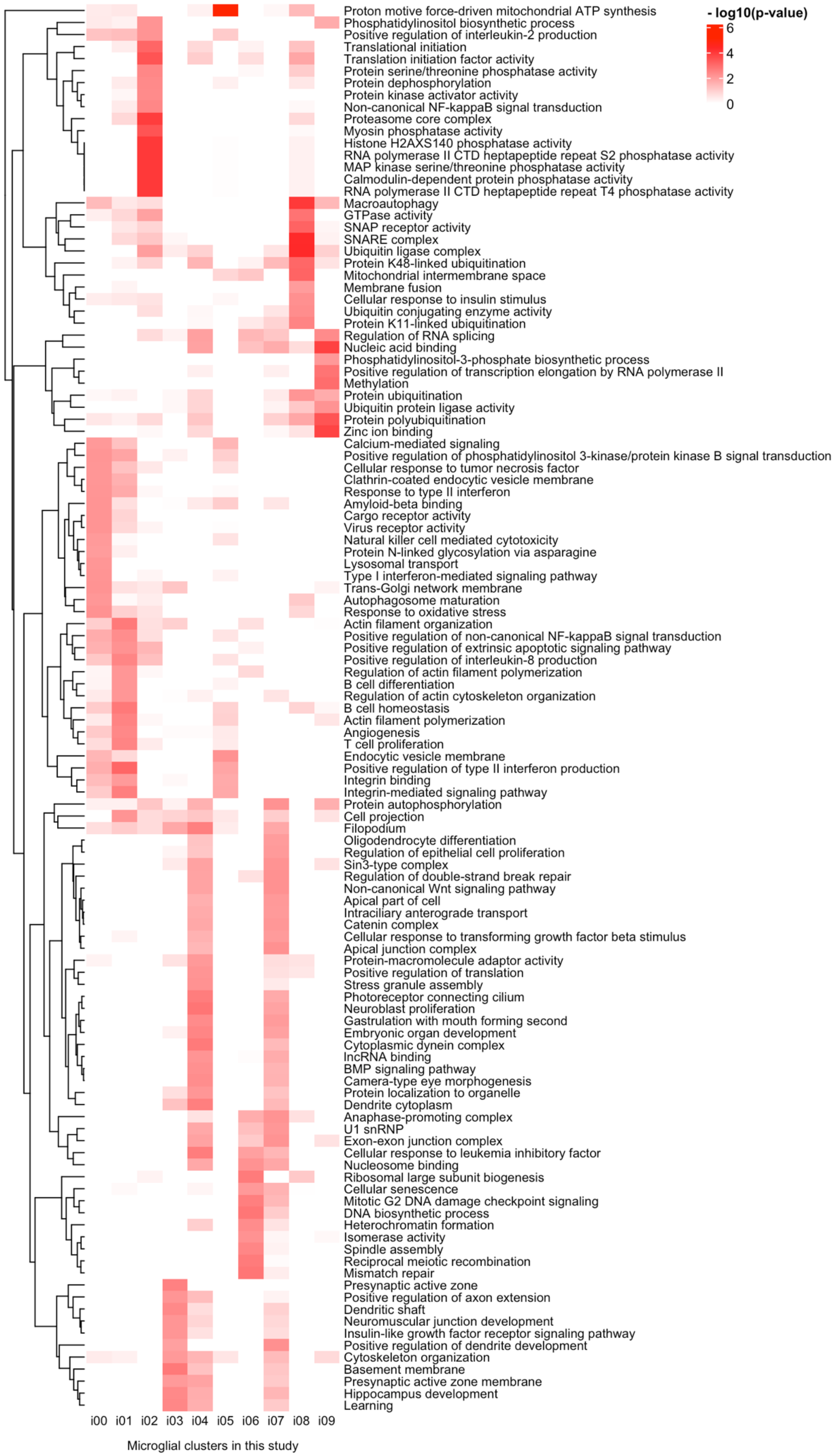

Supplement: Supplementary file 1 — Table S1. Cell fraction of each cell type in iPSC‐derived tri‐cultures in different condition by scRNA‐seq. Fig. S1. Enriched gene ontology biological processes in microglial clusters. Fig. S2. Ferroptosis induction does not affect the number of astrocytes in the iPSC tri‐culture. Fig. S3. Enriched gene ontology biological processes in microglial clusters upon ion + RSL3 treatment. [file FEB4-16-1060-s001.pdf]
